# The Effect of Zhongyong Thinking on Remote Association Thinking: An EEG Study

**DOI:** 10.3389/fpsyg.2019.00207

**Published:** 2019-02-07

**Authors:** Zhijin Zhou, Lixia Hu, Cuicui Sun, Mingzhu Li, Fang Guo, Qingbai Zhao

**Affiliations:** ^1^School of Psychology, Central China Normal University, Wuhan, China; ^2^Special Education Research and Guidance Center, Haidian Education, Beijing, China

**Keywords:** Confucianism, zhongyong thinking, integrated thinking, eclectic thinking, creative problem-solving

## Abstract

The Doctrine of the Mean (zhongyong) introduced by Confucianism is not only an aspect of faith, but also a way of thinking for Chinese individuals. Zhongyong includes two thinking forms: eclectic thinking (ET; i.e., “neither-A-nor-B”) and integrated thinking (IT; i.e., “both-A-and-B”). Given the inclination of Asian individuals toward situational cognition, this study used questions about situations familiar to Chinese undergraduates to activate either ET or IT. This was done to investigate the effects of the two divergent thinking forms of zhongyong on performance levels on the Remote Associates Test (RAT). Both behavioral and EEG results found that participants in the IT condition demonstrated higher RAT scores than those in the ET condition. The conclusion was that the RAT and priming tasks shared the same neural mechanism. This meant that the priming tasks of IT allowed participants to enter a state of creative preparation in advance, further affecting resolution of the RAT.

## Introduction

Creativity is one of the most complex and elusive human behaviors (Agnoli et al., 2018). Exploring the creativity of individuals or groups are gaining momentum across different scientific disciplines. Csikszentmihalyi (1999) argued that creativity is not only a psychological process but also a cultural and social phenomenon. Cultural values influence one’s way of thinking and can have a significant impact on their creativity (Liu et al., 2015). It is well known that Chinese individuals who are influenced by Confucianism have long abided the Doctrine of the Mean (i.e., zhongyong) that provides the third perspectives to settle differences (Pang, 1980, 2000; Zhou, 1994). Zhongyong is the basic principle that Chinese people use to confront the world. It allows them to capture the essence of social operation (Yang, 2014). Therefore, examining the influence of zhongyong on creativity can reveal how cultural factors and principles affect creativity. This research attempted to redefine the concept of zhongyong by exploring its structure based on previous literature on zhongyong. This redefined concept could potentially deepen the understanding of how Chinese traditional culture influences creativity. This study used an experimental method with priming technology and cognitive neuroscience research methods. This could provide researchers with possible neural evidence of the effect of zhongyong on creativity and address the gap in the psychological research of zhongyong.

Zhongyong was initially considered a kind of supreme morality. Over time, the concept has evolved into a value system and overall way of thinking, which focuses on being free from excess/deficiency (i.e., “just right”); however, the concept of zhongyong should not be equated with compromise or egalitarianism (Feng, 1940). Many researchers believe that zhongyong is the basic principle used by the Chinese to confront the world, solve problems, and achieve harmony (Yang and Zhao, 1997; Yang, 2009). The characterization of zhongyong as both mastering extremes, but deploying the mean is part of its dichotomous (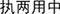) epistemology. In support of this characterization, Pang (1980, 2000) indicated that zhongyong is not only part of the ethics of the Confucianism, but also how the Chinese understand and interact with the rest of the world. There are four forms of zhongyong: “A and B,” which can be conceptualized as being based on A, but with B taken into account, e.g., “the master was mild, and yet dignified,” “warm, yet gentle”(wen’er’li, 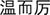), “respectful, yet easy”(gong’er’an, 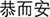); “Both A and B,” which can be conceptualized as including A and B at the same time, such as “being skilled in using both the pen and the rifle”(nengwennengwu, 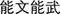) and “both serious and facetious” (yizhuangyixie, 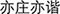); “Neither A nor B,” which can be conceptualized as the opposite of “Both A and B,” such as “neither haughty nor obsequious” (bubeibukang, 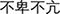), “avoiding leaning to either side” (wupianwupo, 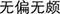); and “A, yet not A’, ” which can be conceptualized as possessing features of A, but with A being prevented from being excessive through the removal of B, such as “majestic without being fierce” (wei’er’bumeng, 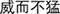), “enjoyment without being immoral”(le’er’buyin, 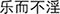). Among the four forms of zhongyong, “A and B” and “both A and B” involve integrative thinking more, while “Neither A nor B” and “A, yet not A”’ favor ET.

The systematic study of zhongyong thinking in psychology began in the late 1990s by Yang and colleagues (Yang and Zhao, 1997; Yang, 2009; Yang and Lin, 2012; Yang et al., 2014). They argued that zhongyong thinking is a “practical thinking system” in which people decide how to choose, execute, and correct specific action plans. However, this “system” proposed by Yang et al. (2014) is overcomplicated, involving values, behaviors, and perceptions related to zhongyong, in which multiple, reciprocating relationships exist among the various components. Therefore, on the basis of this “practical thinking system” of zhongyong constructed by Yang, some researchers began to examine the basic meaning of zhongyong thinking, i.e., “master the extremes, but deploy the mean” (zhiliangduan’er’yunzhong, 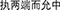). For example, Wu and Lin (2005) focused on cognition and behavior while dealing with controversy or disagreements, and defined zhongyong thinking as “thinking about a problem from multi-perspectives, and making behavioral decisions that take into account both the self and the overall situation after considering different views in detail.” Western researchers have attributed similar concepts to zhongyong thinking, such as cognitive complexity and integrative thinking. However, zhongyong is still more complex than this, composed of integrated, eclectic, and holistic thinking, and changing according to the situation, which represents an important distinction in cognition between Chinese and West.

The method of cognition influences how individuals and groups develop (Ji et al., 2000). The effect of zhongyong on creativity evoked widespread concern among researchers. However, findings on the subject are inconsistent. A study by Zhang and Gu (2015) adopted the Zhongyong Thinking Scale, which includes three dimensions: multi-thinking, holism, and harmoniousness, finding that an employee’s zhongyong thinking score was positively correlated with self-rated creativity. Further, Liao and Dong (2015) found that the zhongyong thinking exerted a positive influence on employee innovation, a relationship partly mediated by organizational harmony. Conversely, some research has found that zhongyong might hinder innovation and the transformation of creative ideas into action (Yao et al., 2010). Liu et al. (2015) utilized the Zhongyong Practical Thinking Scale and the Creative Personality Scale to observe a negative relationship between zhongyong and creative personalities in a group of art majors. We believe the contradictory findings discussed above can be attributed to researchers holding varying concepts of zhongyong, resulting in the use of disparate measurement tools.

Previous research examining zhongyong thinking tended to consider it a static method of thinking, resistant to situation change. However, cross-cultural studies have shown that East Asians practice more situational cognition than Westerners (Ji et al., 2000), paying more attention to background elements of the environment (Nisbett and Miyamoto, 2005), and more likely to attribute elements to the situation (Choi et al., 1999; Morris and Peng, 1994).

As mentioned previously, zhongyong is a combination of various forms of thinking, which can change to suit a given situation. Additionally, Zhou et al. (unpublished) found that integrated thinking (IT), but not eclectic thinking (ET), was associated with significantly higher scores on a Remote Associates Test (RAT), suggesting that the different forms of zhongyong thinking may have different effects on association tasks. These results prompt the question as to whether different forms of zhongyong thinking, applied to different situations, would affect RAT scores and/or neurophysiological measures. In the current study, electroencephalograms (EEGs) were used to record neural activity during various cognitive tasks. EEG designs are an appropriate method to obtain insight in the temporal evolution of cognitive processes (Srinivasan, 2007), allowing for a much more detailed look at brain activation, which can be observed in response to particular cognitive events (e.g., immediately prior to the production of an original idea). Each frequency band of an EEG is related to specific cognitive functions. For instance, the theta frequency band primarily reflects working memory, while the alpha frequency is related to internal attention and semantic processing. Further, the beta plays an important role in language processing pretreatment. A study of creative thinking utilizing EEG measures can better reveal the underlying neural mechanism. Additionally, EEG data contain many different aspects, such as event related synchronization/desynchronization (ERS/D) and task-related power (TRP), which can be used to calculate the coherence of the brain regions, to compare brain activity between the task/baseline states, or to compare differences in neural activity between general and creative thinking.

At present, creative thinking research utilizing EEG primarily focuses on comparing differences in neural activation triggered by creative and general thinking. For instance, Fink et al. (2007, 2009) found that individuals demonstrate increased alpha waves when performing alternative use tasks (AUTs) when compared to general tasks (e.g., completing word suffixes, object feature generation). This indicates top-down activity or, more specifically, selective inhibition of specific brain regions. Mölle et al. (1999) investigated differences in neuroelectrophysiology between divergent and convergent thinking with EEG, and found that the dimensional complexity of EEG signals was greater during divergent thinking, possibly the result of the concurrent activation of a greater number of independently oscillating processing units. However, theta activity in the frontal lobe decreased during the divergent thinking task and increased convergent thinking task. Further, frontal alpha and beta wave activity during both divergent and convergent thinking tasks was lower compared to the control condition. Similarly, Razumnikova (2007) found widespread enhancement of power and coherence in the beta 2 band, increased desynchronization of alpha 1 and alpha 2 over the posterior cortex, and increased the theta 1 power in the frontal cortex in the RAT, when compared to the Simple Associates Task (SAT). To avoid differences caused by the task itself, some researchers have explored the neural activity of creative thinking through different task requirements (i.e., creative vs. general solutions) in the same task. For example, Jauk et al. (2012) examined EEG brain activation related to convergent vs. divergent modes of thinking within the same task and found increased desynchronization of alpha waves in the frontal cortex during general thinking and synchronization of alpha waves in the same region during divergent thinking. In addition, there were some researchers who tried to explore the cognitive process of creativity in natural situations rather than in controlled laboratory settings. For example, Shen et al. (2018) assessed the implicit conceptual structure of everyday insight in diverse naturalistic settings by collecting participants’ descriptions of everyday insight experiences and found that insight experience was a multidimensional construct involving positive affect at the moment of insight, phenomenological experiences relating to the dynamic insight process, solution-related cognitive responses, and post insight reflections.

In summary, previous studies have typically focused on characteristics of EEG power in creative tasks by comparing divergent and convergent thinking, or creative and general thinking. However, this may not be generalizable to the real-world, as individuals are often inspired by others’ ideas or methods, and then carry out creative tasks. Fink et al. (2011) explored whether creativity could be improved by exposure to others’ perspectives, and found that participants who received cognitive interventions performed better in the AUT, with synchronization of alpha waves in the right hemisphere, and weak desynchronization of alpha waves in the parietal and temporal lobes of the left hemisphere. Another recent study examined the impact of neurofeedback training with creative thinking-related brain activities on creativity. Results of this study indicate that an increase in brain activity related to divergent thinking (i.e., alpha and beta waves located in the right parietal lobe) can improve creativity (Agnoli et al., 2018).

The current study was performed in a sample of Chinese college students, who were deeply influenced by Chinese traditional culture, and focused on the neural mechanisms of two different forms of zhongyong thinking on creative problem-solving. The two forms of zhongyong thinking (IT and ET) were primed through the use of different story scenarios. Integrated thinking in this study was in the form of “both A and B,” reflecting the idea of “harmony,” while ET was in the form of “Neither A nor B,” reflecting the idea of “the mean.” Participants were then asked to complete corresponding creative cognitive tasks. During the priming and creative cognitive task period, EEGs of participants were recorded to monitor and evaluate neural activation. Based on the results of previous research, it was hypothesized that IT would improve RAT scores when compared to ET. Further, the current study sought to explore neural evidence of the possible underlying mechanisms responsible for any differences observed.

## Materials and Methods

### Participants

The participants of the study included 36 Chinese college students (19 men, 17 women; mean age 22 years, range 19–26 years). All participants were right-handed, native speakers of Chinese, neurologically healthy by self-report, and normal or corrected-to-normal vision. The participants had no prior experience with RAT or any other similar tests. Participants volunteered to be part of the experiment, provided written informed consent prior beginning the experiment, and received payment following participation. Some data were eliminated from EEG analyses owing to excess artifacts or low superimposition times. In total, data from 31 participants were analyzed for the presence of theta (4–8 Hz), alpha (8–12 Hz), and beta (12–30 Hz) waves in the priming phase, and data from 31 participants were analyzed for the same frequency bands in the RAT phase. Data from 31 participants also were included in behavior analyses.

### Experimental Materials

#### Priming Materials

Sixteen social problems familiar to participants were collected, and each problem was written into a vignette to be solved. The compilation of priming vignettes of IT/ET was based on the meaning of zhongyong (Both A and B, Neither A nor B) proposed by Pang (1980). Two hundred and twenty-three undergraduates participated in completing solutions to social problems. Three psychology postgraduate students were invited to evaluate the solutions to social problems proposed by participants. The evaluators divided responses into IT and ET, taking one side, or invalid according to the definition and characteristics of IT (e.g., foresight, integration, “big picture”) and ET. If more than 85% of participants proposed solutions that used ET/IT to solve the problem in the corresponding priming, the vignette of ET/IT was considered as appropriate priming material. According to the above criteria, a total of six vignettes with good priming effect were obtained and adopted in this study after four rounds of test and revision, including three eclectic thinking priming vignettes and three integrative thinking priming vignettes (one for exercise, two for the formal experiment).

Priming ET requires the subject to stand on the side of an outsider to think about the question. For example, one of the vignettes used to prime ET was the “traffic accident” problem. In the description of the situation, participants found that the perpetrator and victim were both willing to negotiate the problem (i.e., how to handle the traffic accident) privately, but disagreed on the share of responsibility and the amount of compensation. Participants were asked “How do you solve this problem as an outsider?” and were prompted to provide a solution that was acceptable to both sides. Compared to ET, IT was a more complicated form of thinking. To prime IT thinking style, subjects were required to imagine themselves as experts in a certain field and then solve the given problems. For example, in the case of “business in the Internet era,” two people plan to jointly run clothing (sales) business. One wished to run a physical store and the other wants to run an online shop. They have discussed the problem many times, but have failed to reach an agreement. Participants were asked, “How would you solve this problem if you were a successful clothing merchant with sales experience?”.

#### Creative Task

It’s generally believed that creativity theories do not support the measurement of creativity through a single technique (Gong et al., 2016). Instead, they adopt several tests to measure creativity (Mehta and Zhu, 2009). Zhou et al. (unpublished) with three different types of creativity tasks, an AUT, RAT, and insight problems (i.e., riddles) adopted the priming method to explore the effect of zhongyong on creative problem-solving. Results showed that priming only demonstrated a significant impact on RAT scores. Therefore, a self-compiled Chinese RAT was used in this study to measure the creative problem-solving of subjects. Our Chinese RAT is a variant of the English-language RAT developed by Mednick (1968), in which participants are typically presented with three clue words (two-character words) and then participants are asked to provide a fourth target word (two-character word), which can establish a semantic connection with the first three clue words. The three clue words given to the participants would be given to establish a semantic connection and act as a clue for the fourth word. For example, if the three given clue words are baby (ying’er, 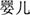), glass (boli, 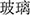), and experiment (shiyan, 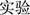), a correct answer would be test tube (shiguan, 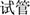); if the given words are invention (faming, 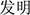), powder (fenmo, 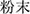), fireworks (yanhua, 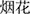), a correct answer would be gunpowder (huoyao, 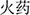). Thirty-six RAT items suitable for college students were used (18 items were used in each experiment) in the current study. Our Chinese version has been validated and has been used in research with native Chinese participants (Zhou et al., unpublished). The difficulty of RAT items ranged from 0.5 to 0.65 in a sample of 110 undergraduates.

### Experimental Procedure

A within-subjects design was adopted in this study and each subject came to the laboratory twice. Participants were randomly allocated to one of two separate priming conditions randomly (i.e., ET priming condition and IT priming condition), with an interval of 5–7 days between sessions. The participants were automatically assigned to the “other” priming condition for the second experiment.

**FIGURE 1:**
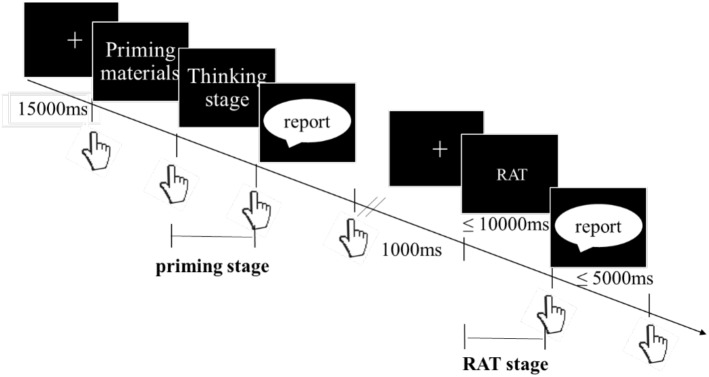
The procedure of a block in the formal experiment. Each experimental condition contains two blocks, each block has 1 priming task and 9 RATs.

Prior to the experiment, all participants were asked to verbally report whether they had prior experience with participating in a RAT test or other similar tests. Then, participants who reported to have no prior experience were asked to rest for 2 min, with eyes open for 1 min and closed for 1 min. EEG data of the resting state (eyes open) were recorded during this period. Participants were then given instructions. A short practice exercise followed to familiarize participants with the experimental process, task requirements, and precautions, such as avoiding head movements, facial expressions, grinding teeth, and swallowing, which may affect EEG data collection. The priming effect was also tested during this period. In the practice phase, participants were presented with the correct answers after answering the first priming question to understand what how they were expected to respond. Data collected during this phase were not used for analysis. When the participants were ready, they would begin the formal experiment. Participants were first presented with a problem situation (vignette) used to activate ET or IT, and then were asked complete a RAT (nine items) while EEG measures were recorded. Participants answered questions orally, and the responses were audio recorded. After a 2-min break, participants were asked to read and complete another zhongyong priming question, followed by another creative thinking task (nine items). The experimental program was administered via E-Prime 2.0. As depicted in Figure 1, each trial started with the presentation of a fixation cross in the middle of the screen for a duration of 15 s (i.e., reference phase). Then, priming material was presented. The subject was asked to read the material quietly, and to press “ENTER” when a solution was reached, reporting the solution orally. In the following RAT phase, participants were shown a fixation point for 1 s. Then, three cue words appeared in the center of the screen, for a duration of 10 s after the fixation point disappeared. Subjects read the cue words as soon as possible and freely associated target words that held a semantic connection with the first three cue words. Participants would push a response button as soon as they believed that they had reached their answer, and the target word was then orally reported. The interface would then begin requesting target words to be reported automatically after 10 s. After this, participants were asked to orally report the target word after 5 s and pressed the “ENTER” button to move to the next question. Failure to answer within the given time limit would be considered a false answer. Data were analyzed with frequency analysis.

### EEG Recording and Analyses

EEG was measured with a 64-channel stretchable electrode cap (Brain Products, Gilching, Germany). The ground electrode and the reference electrode were located at FCz and AFz, respectively. To register eye movements, vertical and horizontal electrooculograms (EOGs) were recorded bipolarly between two electrodes placed diagonally above and below the inner and the outer canthus of the right eye. Electrode impedances were kept below 20 kΩ for the EEG and below 10 kΩ for the EOG. The EEG signals were amplified and bandpass filtered between 0.05 and 100 Hz. All signals were sampled at a frequency of 500 Hz. EEG data were re-referenced by the average signals of Tp9 and Tp10, which were located at left and right ear mastoids, respectively. Analyzer 2.0 software was used to semi-automatically check data for artifacts and artifactual epochs caused by eye blinks, eye movements, or muscle tension. Such anomalies were excluded from further analysis. Band power values (μV^2^) were obtained by squaring filtered EEG signals, and extracting the corresponding frequencies (theta frequency band, 4–8 Hz; alpha frequency band 8–12 Hz; beta frequency band, 12–30 Hz).

As in a previous study (Benedek et al., 2011), the current study analyzed three sections of EEG data: (1) Reference stage-fixation cross before the start of each experiment; (2) Priming stage-when participants were thinking about the problem that was presented; (3) RAT stage-when participants were thinking about the fourth target word (Figure 1). To investigate the process of creative problem solving within different forms of zhongyong thinking, and to reduce the interference of artifacts, data from each stage were segmented and averaged in units of 4 s. These segments were then used in the subsequent analysis of data in each stage.

Brain activity during the performance of experimental tasks was quantified by means of TRP changes in the EEG (Pfurtscheller, 1999; Pfurtscheller and Lopes da Silva, 1999). The TRP for each electrode position was computed by subtracting the log-transformed power during prestimulus reference intervals (Pow_i_,_reference_) from the log-transformed power during the activation intervals (Pow_i_,_activation_) according to the formula: TRP(i) = log[Pow_i_,_reference_]-log[Pow_i_,_activation_]. Therefore, negative values indicate decreases in TRP from the reference to the activation period (i.e., desynchronization), while positive values reflect increases in TRP (i.e., synchronization; Pfurtscheller and Lopes da Silva, 1999). For statistical analyses, electrode positions were topographically aggregated as following: frontal left (AF3, AF7, F3, F5, F7), temporal left (FT7, T7, TP7), parietal left (CP1, CP3, P1, P3), and analogously for the right hemisphere.

## Results

### Behavioral Results

Accuracy rate (i.e., the relative amount of correct responses) and response time (i.e., time until pressing the “ENTER” button in correct trials) were analyzed by means of a repeated measures analysis of variance (ANOVA). Within-subject factors were IT vs. ET. The ANOVA revealed a significant main effect of accuracy rate [*F*(1,30) = 2.986, *p* = 0.006, $\eta_{p}^{2}$ = 0.229], with the accuracy rate in the IT condition (*M* = 0.710, *SD* = 0.168) significantly higher than that observed in the ET condition (*M* = 0.620, *SD* = 0.152). Additionally, it was observed that response time of participants was marginally significantly faster [*F*(1,30) = -1.771, *p* = 0.087, $\eta_{p}^{2}$ = 0.095] in the IT condition (*M* = 4.480, *SD* = 1.137) than in the ET condition (*M* = 4.880, *SD* = 0.840).

### EEG Results

**FIGURE 2:**
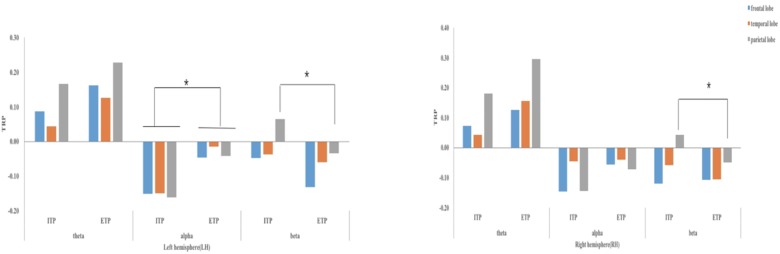
Task-related power of EEG bands (theta, alpha, and beta) in different areas of LH and RH (frontal lobe, temporal lobe, and parietal lobe) in priming stage. ^∗^*p* < 0.05; TRP, task-related power; ITP, integrated thinking problems; ETP, eclectic thinking problems.

A repeated measures ANOVA was performed for TRPs in the theta (4–8 Hz), alpha (8–12 Hz), and beta (12–30 Hz) bands priming condition (IT vs. ET), cerebral hemisphere (left vs. right), and area of the brain (frontal, temporal, parietal) as within-subjects variables. ANOVA revealed significant main effect of priming in the alpha band [*F*(1,30) = 4.772, *p* = 0.038, $\eta_{p}^{2}$= 0.136]. The main effect of priming condition indicates that desynchronization of alpha in the IT condition is higher than ET condition. The ANOVA further revealed significant triple interactions between priming condition, area, and hemisphere in the alpha band [*F*(2,60) = 3.215, *p* = 0.047, $\eta_{p}^{2}$ = 0.097]. The simple effects showed that desynchronization of alpha in the IT condition is higher than ET condition in the left frontal, left temporal and left parietal lobes. The ANOVA further revealed significant triple interactions between priming condition, area, and hemisphere in the beta band [*F*(2,60) = 3.430, *p* = 0.039, $\eta_{p}^{2}$ = 0.103], suggesting beta synchronization in the IT condition and beta desynchronization in the ET condition in bilateral parietal lobes. However, differences in theta power were not found to be significant among areas, hemispheres, or priming conditions. The synchronization or desynchronization of theta, alpha, and beta in IT and ET conditions is shown in Figure 2.

**FIGURE 3:**
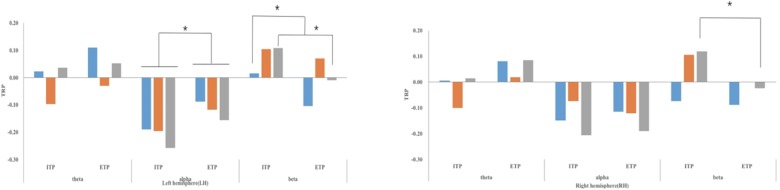
Task-related power of EEG bands (theta, alpha, and beta) in different areas of LH and RH (frontal lobe, temporal lobe, and parietal lobe) in RAT stage. ^∗^*p* < 0.05; TRP, task-related power; CIT, condition of integrated thinking; CET, condition of eclectic thinking.

In the RAT stage, ANOVA revealed a significant double interaction between priming condition and hemisphere in the alpha band [*F*(1,30) = 8.912, *p* = 0.006, $\eta_{p}^{2}$ = 0.229]. Specifically, the alpha desynchronization in the IT condition was significantly higher than in the ET condition in the left hemisphere, yet was not significant in the right hemisphere. In addition, analysis revealed significant triple interactions between priming condition, area, and hemisphere in the beta band [*F*(1.586,47.568) = 4.816, *p* = 0.018, $\eta_{p}^{2}$ = 0.138]. Further simple effect analysis showed the IT priming was associated with increased beta synchronization, while ET priming tended to increase beta desynchronization in the left frontal and bilateral parietal lobes. Like the priming stage, there was also no significant difference of theta wave power among the three conditions in the RAT stage. Corresponding EEG results are shown in Figure 2, 3.

## Discussion

This study investigated the impact of different forms of zhongyong thinking closely related to Chinese traditional culture on creative problem-solving. Based on previous research, zhongyong thinking was divided into IT and ET in the current study. Priming paradigms were adopted to induce participants’ ET or IT. Participants were first presented with a real-world problem situation which needed to be solved with ET or IT. Then participants were asked to complete a RAT. Finally, the performance of participants on the RAT was examined in relation to priming conditions.

Behavioral findings revealed higher accuracy and shorter response time in IT priming than in ET priming, which may be related to different characteristics of different forms of zhongyong thinking. IT not only involves encoding information and retrieving information from long-term memory but also requires participants to integrate information from various sources. In comparison, ET involves less information integration than is typically required within a RAT, such that an individual can solve problems simply based on the retrieval and encoding of information. Additionally, the minds of participants were likely in a heightened integrative state following IT priming, making it easier to link concepts together in the RAT phase, resulting in improved performance. Therefore, results of the current study indicate that IT is more conducive to creative problem solving when compared to ET. These results also indirectly provide evidence that the testing protocol of the current study was effective in manipulating different forms of zhongyong thinking.

In the priming stage, EEG results showed that alpha desynchronization in the IT condition was significantly higher than in the ET condition in the left frontal, left temporal and left parietal lobes. Some studies have found that the phenomenon of alpha desynchronization is observed as a function of increased cognitive load (Stipacek et al., 2003; Fink et al., 2005). As previously mentioned, IT not only involves ET’ mainly function – encoding information and retrieving information from long-term memory, but also requires to integrate information from various sources. Thus, IT may require more cognitive resources. It was found that IT was associated with beta synchronization, while ET was related to beta desynchronization in the bilateral parietal lobes. Some researchers have argued that synchronization in beta waves represents attentiveness and a binding mechanism that subserves perceptual and cognitive functions (Von and Sarnthein, 2000). Additionally, Engel and Fries (2010) have also pointed out that tasks involving endogenous top-down processes are often accompanied by increases in beta power. The IT aspect of zhongyong focuses on integrating information from different sources, which may require top-down processes, such as cognitive control and attention (Bhattacharya and Petsche, 2005; Fink and Benedek, 2014; Schwab et al., 2014). Conversely, unlike IT, ET mainly encodes and retrieves information, and lacks an information integration function. As such, beta desynchronization reflects the encoding of large amounts of external information, as well as the encoding and retrieval of long-term memory information, rather than the integration of information (Weiss and Mueller, 2012), which is essentially the function of ET. Therefore, IT involves more top-down processes, while ET only involves the retrieving and encoding of information in the priming stage.

In the RAT, EEG results showed that alpha desynchronization in the IT condition was significantly higher than in the ET condition in the left hemisphere. IT engages not only information integration but also the retrieving and encoding information. Thus, it may require more cognitive resources. Some studies have found that the phenomenon of alpha desynchronization is typically observed during performance of conventional cognitive tasks, and an increase of alpha band ERD is observed as a function of increased cognitive load (Stipacek et al., 2003; Fink et al., 2005). This study also found IT priming was associated with increased beta synchronization, while ET priming tended to increase beta desynchronization in the left frontal and bilateral parietal lobes. Many studies have indicated the temporal and frontal lobes are key brain regions for solving semantic creativity problems (Jung-Beeman et al., 2004; Qiu et al., 2010; Zhao et al., 2013; Shen et al., 2017). Further, a significant increase of beta activity over the parietal region is associated with better RAT performance, likely reflecting enhanced attentiveness and binding capacity (Bhattacharya and Petsche, 2005; Razumnikova, 2007). These results may be observed because a RAT mainly examines the abilities of word association and concept integration. IT is involved in top-down processes to integrate information, which is the exact skill necessary to excel on a RAT. In contrast, ET is primarily associated with information retrieval and encoding. While these functions are necessary to solve RAT problems, the top-down processes of information integration that ET lacks are also necessary for the final stages of solving a RAT.

On the whole, results found that EEG activity in the RAT phase was similar to that observed in the priming phase. Especially, IT and ET were found to trigger the same EEG activities. Specifically, priming phase and RAT phase both showed beta synchronization and beta desynchronization, respectively, in the bilateral parietal lobes and IT condition with higher alpha desynchronization than ET condition in the left hemisphere. This suggests that RAT and priming tasks might share the same neural mechanism, both of which are likely based on the top-down processing utilized in IT and the retrieval and encoding of information that is a result of ET. Thus, different forms of thinking that are initiated by priming tasks allowed participants to enter into a corresponding state of preparation in advance, further affecting problem-solving ability, as evidenced by RAT performance, which is consistent with the current behavioral findings.

In summary, participants primed in IT showed better RAT performance than those primed for ET, indicating that IT allows for top-down processing of information integration, differing substantially from ET, which appears to focus more on the retrieval and encoding of information. Simultaneously, priming tasks of zhongyong thinking might share a common neural mechanism activated by the RAT, so participants were in an enhanced state of integrative mind preparation after the IT priming, making it easier to integrate and link information in the RAT phase, and further improving RAT performance.

## Limitations

It is worth mentioning that this study utilized only one creative thinking task. This may limit the generalizability of the findings to other creative tasks, such as divergent thinking. However, it is important to note that the top-down process involved in a RAT may be the same as those necessary for other types of creative tasks. Additionally, the EEG method was adopted in this study to explore the cognitive process of the effect of zhongyong thinking priming on RAT. As we know that the EEG has a low spatial resolution, so future research may consider using fMRI (functional magnetic resonance imaging) instead. Last, getting participants to apply zhongyong while solving problems resulted in subjects completing very few priming problems (two problems in each formal experiment). Therefore, some important data (effective EEG signal ) may have drowned out due to fewer overlapping times and high noise during the experiment.

## Ethics Statement

This study is approved by the Ethics Institutional Review Board of Central China Normal University and all study participants provided informed consent. We have read and understood your journal’s policies, and we believe that neither the manuscript nor the study violates any of these.

## Author Contributions

All authors listed have made a substantial, direct and intellectual contribution to the work, and approved it for publication.

## Conflict of Interest Statement

The authors declare that the research was conducted in the absence of any commercial or financial relationships that could be construed as a potential conflict of interest.
